# Ultraviolet exposure of Gafchromic XR‐RV3 and XR‐SP2 films

**DOI:** 10.1120/jacmp.v16i5.5664

**Published:** 2015-09-08

**Authors:** Toshizo Katsuda, Rumi Gotanda, Tatsuhiro Gotanda, Takuya Akagawa, Nobuyoshi Tanki, Tadao Kuwano, Kouichi Yabunaka

**Affiliations:** ^1^ Faculty of Human Relation Tokai Gakuin University Kakamigahara; ^2^ Department of Radiological Sciences Ibaraki Prefectural University of Health Sciences Ibaraki; ^3^ Faculty of Health Sciences Junshin Gakuen University Fukuoka; ^4^ Department of Radiological Technology Tokushima Red Cross Hospital Tokushima; ^5^ Center for Life Science Technologies RIKEN Kobe; ^6^ Graduate School of Health Sciences Okayama University Okayama; ^7^ Graduate School of Medicine the University of Tokyo Tokyo Japan

**Keywords:** ultraviolet rays, nonuniformity, Gafchromic film, high‐resolution measurement, computed tomography

## Abstract

Gafchromic film has been used for X‐ray dose measurement in diagnostic examinations. Their use has been initiated for three‐dimensional X‐ray dose measurement by using the high‐resolution characteristics of Gafchromic films in computed tomography. However, it is necessary to solve the problem of nonuniform thickness in the active layers of Gafchromic films. A double exposure technique using X‐rays is performed in therapeutic radiology; it is difficult to use in a diagnostic examination because of the heel effect. Therefore, it is suggested that ultraviolet (UV) rays be substituted for X‐rays. However, the appropriate UV wavelength is unknown. In this study, we aimed to determine which UV wavelengths are effective to expose Gafchromic XR‐RV3 and XR‐SP2. UV lamps with peak wavelengths of 245 nm, 310 nm, and 365 nm were used. The three UV wavelengths were used to irradiate Gafchromic XR‐RV3 and XR‐SP2 films for 60 min, and irradiation was repeated every 60 min for 600 min thereafter. Films were scanned after each irradiation period on a flatbed scanner. The images were split into their red‐green‐blue components, and red images were stored using ImageJ version 1.44o image analysis software. Regions of interest (ROI), 0.5 inches in diameter, were placed at the centers of the subtracted Gafchromic film images, and graphs of UV irradiation duration and mean pixel values were plotted. There were reactions to UV‐A on both Gafchromic XR‐RV3 and XR‐SP2; those to UV‐B were moderate. However, UV‐C demonstrated few reactions with Gafchromic XR‐RV3 and XR‐SP2. From these results, irradiation with UV‐A may be able to correct nonuniformity errors. Uniform UV‐A irradiation of Gafchromic films with large areas is possible, and UV rays can be used as a substitute for X‐rays in the double exposure technique.

PACS number: 87.53 Bn

## I. INTRODUCTION

Gafchromic film has been used for the measurement of X‐rays in diagnostic radiology applications such as computed tomography (CT), interventional radiology (IVR), quality control (QC), and quality assurance (QA). In the measurement of CT dose, three‐dimensional dosimetry methods have been developed, such as a sheet roll phantom with Gafchromic films^(1)^ and a half cylindrical phantom with Gafchromic films.^(2)^ The uniformity of Gafchromic film is important to ensure a high‐resolution measurement. Nonuniformity errors from Gafchromic films arise due to variation in the thickness of the active layer, which affects the measured dose and adds noise to the data signal from X‐ray exposure. When a change in the density of the Gafchromic film is assumed as a true signal in a real X‐ray, the density inhomogeneity caused by the thickness irregularity of the active layer of the Gafchromic film is presented as noise. If this irregularity is not corrected, noise will be added to the true signal. One correction method for this is a double exposure technique where the film is first irradiated with uniform X‐rays before X‐ray irradiation in small quantities. Thickness irregularity is converted into density data and is used to obtain only true data by subtracting the noise. A double exposure technique is applied to reduce such nonuniformity errors;^(3)^ however, it is difficult to homogeneously expose a large area to X‐rays because of the heel effects from using diagnostic X‐ray device. Zhu et al.^(3)^ have proposed this technique which is applicable to all radiochromic films. If evidence of nonuniformity should appear, the double‐exposure technique is a method to improve film uniformity to an acceptable tolerance.^(4)^ Gafchromic film reacts to different spectrum of ultraviolet (UV) rays and can be used to measure the amount of solar UV rays used in this reaction.^5^, ^6^ Butson et al.^(6)^ put the X‐ray device outdoors and, using Gafchromic EBT2 film, measured UV rays of the light of the sun. This is a measurement of a large quantity of UV rays. It is shown that it can be measured in the quantity of the UV rays in this study.

Irradiating Gafchromic films with UV is not usually recommended^(7)^ as density change of the film by UV rays becomes noise and reduces the precision of the measured value. However, the nonuniformity error arising from the thickness of the Gafchromic film can be expressed as density irregularity by utilizing UV irradiation as a substitute for X‐ray irradiation in the double exposure technique. UV irradiates large areas uniformly, and the nonuniformity error can be reduced by subtraction. We preliminarily exposed UV (360 nm) rays (0.018 mW/cm^2^) to Gafchromic EBT film with the double exposure technique; good results were obtained.^(8)^ True exposure data were obtained by utilizing UV light at 360 nm without actual X‐ray exposure. In other words, data initialization of the Gafchromic EBT is possible. When exposed to equal X‐ray dose to arbitrary segments of Gafchromic EBT, the density data indicate equal value.^(8)^

As nonuniformity in the thickness of the active layer affects the true data as noise, UV was irradiated uniformly as a double exposure with the aim of removing such nonuniformity errors. An effect was confirmed for the homogeneous improvement of UV irradiation on Gafchromic EBT; however, the effect is unknown for Gafchromic XR‐RV3 (lot # 05221406) and XR‐SP2 (lot # 01311401) (Ashland Inc.; Covington, KY).

In addition, these films are constructed to be protected from UV rays. The reactions when UV is irradiated onto Gafchromic XR‐RV3 and XR‐SP2 are unknown. Primarily, the sensitivity of the Gafchromic film active layer to UV rays must be clarifed.

There are various wavelengths of UV rays ranging from 200 nm to 400 nm. The most suitable wavelength or adaptation wavelength needs to be determined. In general, UV rays are divided into UV‐A, UV‐B, and UV‐C depending on wavelength.^(8)^ The wavelengths of UV‐A range from 315 to 400 nm, while those of UV‐B range from 280 nm to 315 nm. The wavelengths of UV‐C range from 200 to 280 nm.^(8)^ In this study, wavelengths of 365 nm, 310 nm, and 245 nm were used for UV‐A, UV‐B, and UV‐C, respectively; the sensitivity of the films to the UV radiation wavelengths were compared because Gafchromic films used in this study were unlike those used in the study by Katsuda et al.^(8)^ Therefore, the focus of this study was to determine suitable UV exposure wavelengths for Gafchromic XR‐RV3 and XR‐SP2 films.

Because Gafchromic XR‐RV3 and XR‐SP2 have UV protection, it is thought that the reaction of UV rays is inferior to those of Gafchromic EBT. Therefore, the distance from the surface of the UV lamp to Gafchromic films was set at 7.5 cm, the irradiation duration was lengthened, and an experiment was conducted.

Therefore, in this study, we aimed to find suitable UV‐A, ‐B, and ‐C rays that are more reactive with Gafchromic XR‐RV3 and XR‐SP2 active layers as a step before attempting the double exposure method using UV on Gafchromic XR‐RV3 and XR‐SP2.

The purpose of this study is to decide optimum UV energy for use as a substitute for X‐ray energy in the double exposure technique.

## II. MATERIALS AND METHODS

### A. Gafchromic films and UV lamps

Two kinds of Gafchromic films were used in this study: Gafchromic XR‐RV3 and XR‐SP2. The Gafchromic XR‐RV3 and XR‐SP2 are UV‐protected films.

The two kinds of Gafchromic film were exposed to three kinds of UV rays. UV lamps with peak wavelengths of 365 nm (UV‐A), 310 nm (UV‐B), and 245 nm (UV‐C) were used. The UV lamp that generated a peak UV wavelength of 365 nm used a black light, NEC FL10SBL (10 W) (NEC Lighting, Ltd.; Tokyo, Japan). The lamp that generated a peak wavelength of 310 nm used a UV‐B chemical lamp, FL‐10E (10 W) (Kyokko Denki Co., Ltd.; Tokyo, Japan). A sterilization lamp that generated a peak UV wavelength of 254 nm was also used, Toshiba GL10SBL (10 W) (Toshiba Lighting & Technology Corporation; Kanagawa, Japan).

### B. UV exposure

**Figure 1:**
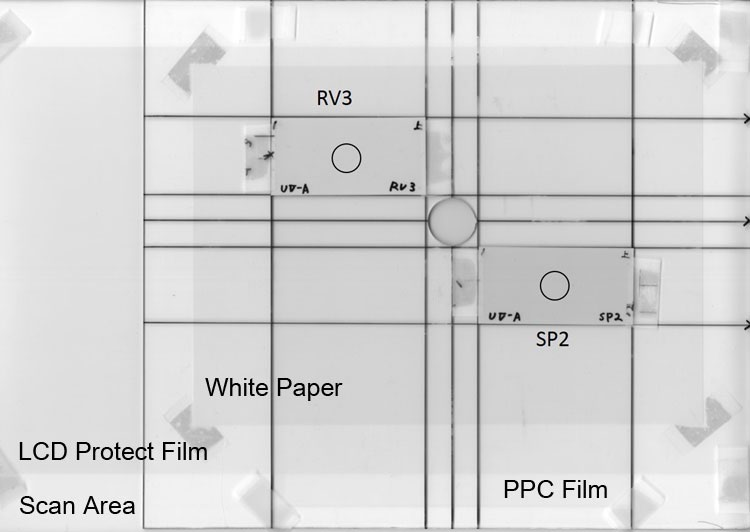
Placements of Gafchromic XR‐SP2 and XR‐RV3. The black circles indicate the measured ROIs for the pixel values.

The experiment was conducted at night to remove the influence of solar UV radiation. The fluorescent lamps in the room were exchanged for UV ray‐cutting lamps. The cutting of Gafchromic films were performed under an incandescent lamp. Using a UV lamp, the surrounding exposure area was covered with UV‐protective acrylic plates (Comogras CG UV40 P 3‐mm thickness Lot # 140406C B, Kuraray Co., Ltd., Tokyo). Gafchromic films were cut by 9 cm on the long axis and 4.5 cm on the short axis. The Gafchromic XR‐SP2 and XR‐RV3 were placed on an acrylic board 3 mm in thickness (Fig. 1).

**Figure 2:**
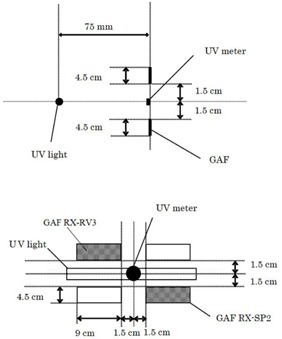
The setup arrangement for ultraviolet (UV) exposure. A lateral view (top) and frontal view (bottom) are shown.

Frontal and lateral views of the arrangements of UV exposure to Gafchromic films are shown in Fig. 2. The distance between the UV source surface and optical receiver surface was 7.5 cm. A UV meter probe was placed at the center of the exposure area, and the strength of the UV irradiation to Gafchromic film was also measured. UV strength was measured using a UVR‐300 with UD‐360 (365 nm) and UD‐250 (245 nm) probes (Topcon Technohouse Corp.; Tokyo, Japan). A UV light meter (UV‐340A; Mother Tool Corporation; Nagano, Japan) was used to measure 310 nm UV rays.

### C. Image scanning

Each of the UV rays were irradiated for 60 min, and the irradiation was repeated every 60 min thereafter for 600 min. UV irradiated Gafchromic XR‐RV3 and XR‐SP2 were scanned after every irradiation on a flatbed scanner (EPSON ES‐10000G; Seiko Epson Co.; Nagano, Japan) without scanner nonuniformity corrections and acquired as an image by using Adobe Photoshop CS2 (Adobe Systems Incorporated; San Jose, CA). It was read at 48 bits and 100 dpi resolution in RGB mode with PPC film (CR‐PP686; 3M Company; St. Paul, MN) and a liquid crystal protection film (LCD‐230W; Sanwa Supply Inc.; Okayama, Japan)^(9)^ for the removal of moiré artifacts (Newton's rings). As the scan orientation is important to the response of Gafchromic films, they were always scanned in the same direction (portrait for Gafchromic XR‐RV3 and landscape for XR‐SP2) according to the uncut film size and the size of the scanner area. The scan position of the scanner is also important, as data varies between the center and the lateral side.^(10)^ Therefore, we always scanned the Gafchromic films in a similar position and orientation, attached to acrylic plates.

The work from the preparations to the scanning of Gafchromic films was performed in a room where the temperature range was limited between 21°C and 25°C.

### D. Image analysis

A ROI 0.5 inches in diameter was placed at the center of the films and the mean pixel value ± standard deviation (SD) was measured (Fig. 1). For the image data, the pixel value was analyzed in ImageJ version 1.44o image analysis software for Macintosh (National Institutes of Health, Bethesda, MD). Graphs of UV exposure time and pixel values were plotted.

## III. RESULTS

The irradiation strengths of UV‐A, UV‐B, and UV‐C were 1.438 mW/cm^2^, 0.994 mW/cm^2^, and 1.761 mW/cm^2^, respectively. The amounts of UV‐A, UV‐B, and UV‐C irradiation over 600 min were 51.77 J/cm^2^, 35.78 J/cm^2^, and 63.40 J/cm^2^, respectively, as shown in Table 1

Mean pixel values ± SD of three spectrum of UV irradiated for 600 min to Gafchromic XR‐RV3 were 1743.88±169.02 (UV‐A), 630.38±111.11 (UV‐B), and 663.86±104.95 (UV‐C). Mean pixel values ± SD of three spectrum of UV irradiated for 600 min to Gafchromic XR‐SP2 were 754.14±148.83 (UV‐A), 227.94±119.11 (UV‐B), and 469.90±159.92 (UV‐C). Results of three spectrum of UV rays irradiated to Gafchromic XR‐RV3 and XR‐SP2 are shown in 3, 4.

When UV‐A was irradiated for 600 min, mean pixel value showed highest value. In the case of UV‐B, there was a moderate reaction on Gafchromic XR‐SP2, but there were few reactions on Gafchromic XR‐RV3. In addition, in the case of UV‐C, both Gafchromic XR‐RV3 and XR‐SP2 demonstrated few reactions.

From these results, it is shown that UV‐A can be used for pre‐irradiation of Gafchromic XR‐RV3 and XR‐SP2. Figure 5 shows the reactions of UV‐A between the Gafchromic XR‐RV3 and XR‐SP2.

**Table 1:** Three kinds of UV rays strength that irradiated Gafchromic films

*Ex Time (min)*	$UV\;Strength(J/cm^{2}=W/cm^{2}\times s)$		
	*UV‐A*	*UV‐B*	*UV‐C*
60	5.18	3.58	6.34
120	10.35	7.16	12.68
180	15.53	10.73	19.02
240	20.71	14.31	25.36
300	25.88	17.89	31.70
360	31.06	21.47	38.04
420	36.24	25.05	44.38
480	41.41	28.63	50.72
540	46.59	32.21	57.06
600	51.77	35.78	63.40

**Figure 3:**
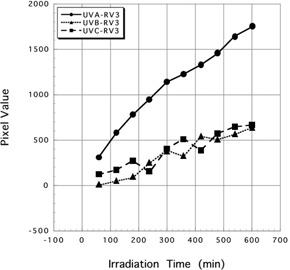
Results of UV‐A, B, and C exposure to Gafchromic XR‐RV3. Marker (black circle, black square, and black triangle) show the scanning timing of each irradiated Gafchromic film.

In other words, for Gafchromic XR‐RV3 and XR‐SP2, it may be said that a reaction is recognized within 60 min of UV‐A exposure (3, 4). The reaction is acceptable to use double exposure substitute to X‐rays.

**Figure 4:**
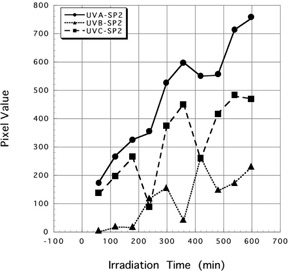
The different reactions of UV‐A between the Gafchromic XR‐RV3 and XR‐SP2. Marker (black circle, black square, and black triangle) show the scanning timing of each irradiated Gafchromic film.

**Figure 5:**
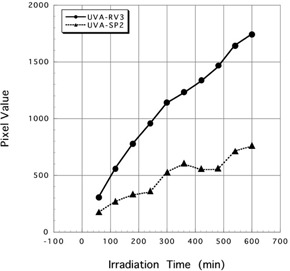
Results of UV‐A, B, and C exposure to Gafchromic XR‐SP2. Marker (black circle, black square, and black triangle) show the scanning timing of each irradiated Gafchromic film.

## IV. DISCUSSION

### A. UV wavelength

In general, the UV spectrum is divided into UV‐A, UV‐B, and UV‐C, depending on wavelength.^(11)^ The UV‐A wavelengths range from 315 to 400 nm, UV‐B wavelengths range from 280 to 315 nm, and UV‐C wavelengths range from 200 to 280 nm.^(11)^ In the present study, it was proven that UV‐A was a satisfactory choice for using UV irradiation substitute to X‐rays for double exposure technique.

### B. Reactions

Both Gafchromic XR‐RV3 and XR‐SP2 are afforded UV protection so as not to be affected by UV rays, and almost did not react to the UV rays of the fluorescent lamps. The purpose of this study was to use UV rays as a substitute for X‐rays for pre‐irradiation prior to the double exposure technique. Therefore, a UV lamp was used, and it was decided that a large amount of UV could be used to irradiate the Gafchromic XR‐RV3 and XR‐SP2. A color change in the active layer of Gafchromic film was difficult to evaluate visually by using 10‐W UV lamps. However, a clear reaction was shown after image processing, and the possibility of using UV for pre‐irradiation prior to the double exposure technique was determined. During this time, 5.1 to 51 J/cm^2^ of UV‐A was irradiated, but the reactions were different between the Gafchromic XR‐RV3 and XR‐SP2 (Fig. 3). The graph of Gafchromic XR‐RV3 shows the rise that is more sudden than a graph of XR‐SP2, and the sensitivity for the UV of Gafchromic XR‐RV3 is clearly higher than XR‐SP2.

### C. UV protection

There is a method to correct nonuniformity error where yellow dye is included in the active layer of Gafchromic EBT2 and EBT3, and signal information is assumed from a red channel, while nonuniformity error information is obtained from a blue channel such that it can be corrected.^12^, ^13^ However, the entire surfaces of Gafchromic XR‐RV3 and XR‐SP2 are orange, and it may be said that the coloration of the active layer with a yellow dye would not be meaningful. Therefore, the double exposure technique and subtraction technique are necessary to correct nonuniformity errors on Gafchromic XR‐RV3 and XR‐SP2.

### D. UV protection for humans

The wavelengths of UV‐C and UV‐B are short and affect the human body.^14^, ^15^ A UV irradiation box was constructed from an acrylic board to reduce UV exposure for the experimenter. There was no leakage of UV rays from inside the box.

### E. Future study

We used UV‐A fluorescent tube types, so‐called black lights. However, the wavelength of UB‐A varies in the range 315–400 nm and it is unknown which wavelengths were most efficient. It will be necessary to investigate this issue further with a lamp producing UV rays at wavelengths in a small range. An experiment using UV ray outbreak apparatuses (e.g., LEDs) that generate specific wavelengths is necessary to identify the optimal wavelength.

## V. CONCLUSIONS

The reactions to UV were checked to determine whether a double exposure technique using UV could be applicable to Gafchromic XR‐RV3 and XR‐SP2. A density increase that resulted in difficulties in judgment when viewing was recognized by performing an image analysis and irradiating both Gafchromic films with large quantities of UV‐A. A rise in this density could possibly correct uncertainties due to any nonuniform of the thickness in the active layer.

## ACKNOWLEDGMENTS

This study was supported by JSPS KAKENHI Grant Numbers 26460740.

## Supporting information

Supplementary Material
